# Gaps in communication theory paradigms when conducting implementation science research: qualitative observations from interviews with administrators, implementors, and evaluators of rural health programs

**DOI:** 10.1186/s13012-024-01395-3

**Published:** 2024-09-16

**Authors:** Nicole L. Johnson, Jennifer Van Tiem, Erin Balkenende, DeShauna Jones, Julia E. Friberg, Emily E. Chasco, Jane Moeckli, Kenda S. Steffensmeier, Melissa J. A. Steffen, Kanika Arora, Borsika A. Rabin, Heather Schacht Reisinger

**Affiliations:** 1 Center for Access and Delivery Research and Evaluation, Iowa City VA Healthcare System, Iowa City, IA USA; 2 Veterans Rural Health Resource Center-Iowa City (VRHRC-Iowa City), VA Office of Rural Health, Iowa City, IA USA; 3 Division of General Internal Medicine, Department of Internal Medicine, Carver College of Medicine, University of Iowa, Iowa City, IA USA; 4 Institute for Clinical and Translational Science, University of Iowa, Iowa City, IA USA; 5 Department of Health Management and Policy, College of Public Health, University of Iowa, Iowa City, IA USA; 6 Herbert Wertheim School of Public Health and Human Longevity Science, University of California San Diego, La Jolla, CA San Diego, USA; 7 UC San Diego ACTRI Dissemination and Implementation Science Center, University of California San Diego, La Jolla, CA San Diego, USA

**Keywords:** Leadership buy-in, Collaboration, Interviewing, Qualitative methods, Communication theory, Veteran, Implementation science strategies

## Abstract

**Background:**

Communication is considered an inherent element of nearly every implementation strategy. Often it is seen as a means for imparting new information between stakeholders, representing a Transaction orientation to communication. From a Process orientation, communication is more than information-exchange and is acknowledged as being shaped by (and shaping) the individuals involved and their relationships with one another. As the field of Implementation Science (IS) works to strengthen theoretical integration, we encourage an interdisciplinary approach that engages communication theory to develop richer understanding of strategies and determinants of practice.

**Methods:**

We interviewed 28 evaluators, 12 implementors, and 12 administrators from 21 Enterprise-Wide Initiatives funded by the Department of Veteran Affairs Office of Rural Health. Semi-structured interviews focused on experiences with implementation and evaluation strategies. We analyzed the interviews using thematic analysis identifying a range of IS constructs. Then we deductively classified those segments based on a Transaction or Process orientation to communication.

**Results:**

We organized findings using the two IS constructs most commonly discussed in interviews: Collaboration and Leadership Buy-in. The majority of segments coded as Collaboration (*n* = 34, 74%) and Leadership Buy-in (*n* = 31, 70%) discussed communication from a Transaction orientation and referred to communication as synonymous with information exchange, which emphasizes the task over the relationships between the individuals performing the tasks. Conversely, when participants discussed Collaboration and Leadership Buy-in from a Process orientation, they acknowledged both constructs as the result of long-term efforts to develop positive relationships based on trust and respect, and emphasized the time costliness of such strategies. Our findings demonstrate that participants who discussed communication from a Process orientation recognized the nuance and complexity of interpersonal interactions, particularly in the context of IS.

**Conclusions:**

Efficient, reliable information exchange is a critical but often overemphasized element of implementation. Practitioners and researchers must recognize and incorporate the larger role of communication in IS. Two suggestions for engaging a Process orientation to communication are to: (a) use interview probes to learn how communication is enacted, and (b) use process-oriented communication theories to develop interventions and evaluation tools.

**Supplementary Information:**

The online version contains supplementary material available at 10.1186/s13012-024-01395-3.

Contributions to the literature

- Communication is a vital part of implementation. Yet, predominant discussions about implementation strategies are limited to a Transactional orientation. Conversely, the Process orientation to communication acknowledges the multiple moving elements in an implementation context that influences collaboration and leadership buy-in.
- Exemplars of interview segments about communication engaging a Process orientation were identified to demonstrate ways interviewers can probe to gain a deeper understanding of communication as a process.
- We provide examples and suggestions for qualitatively examining communication processes to better understand the impact of implementation strategies.
- Several theories with a Process orientation are identified for consideration in future research and implementation planning and evaluation.

## Background

Most implementation strategies include a communication component, particularly when evidence-based interventions are introduced and promoted throughout an organization. When implementing new programming, it is common to consider communication as simply a means through which information is imparted [1, 2]. Implementation Science (IS) researchers have an imperative to understand the role of communication as more than a means for information exchange [3]. Yet, even as a means for information exchange, Manojlovich and colleagues recognized the lack of attention on communication in implementation research [1].

Broadly, the study of communication focuses on how messages are used to generate meanings [4], and provides perspective for moving beyond an emphasis on information exchange, thus moving beyond the task dimension and recognizing the value of the relational dimension. Despite its relatively young development both academically and professionally, the communication discipline offers valuable insight to IS research [5]. There are two predominant ways to characterize communication: (1) communication as Transaction, and (2) communication as Process. When communication is viewed as a Transaction, it is discussed as a linear one-way flow of information [3]. The materiality – the element of substantive value – of communication is found in accurate, efficient information transfer, thus putting emphasis on the task dimension and channel (e.g., phone, handout) through which information is exchanged. When practitioners focus their efforts on preparing thoughtful and detailed educational sessions intended to increase program adoption, but do not allow time for interactive questions or develop opportunities for building relationships between key personnel responsible for successful adoption, then we see a reliance on the Transaction orientation to communication. When communication is conceptualized as a Process, we emphasize its constitutive nature wherein our environments – social, organizational, political, etc. – shape and are shaped through communication [3]. From a Process orientation, the transformative properties of communication emphasize its relational dimension and bring about a materiality from the intangible elements of the process (e.g., tone of voice, relational history, contextual exigency), and concepts such as psychological safety, mutual respect, and trust foreground the mechanics of information exchange. For example, someone may schedule multiple options for the same information session to ensure real-time interactivity for questions and build in opportunities for small group breakouts and post-presentation networking for relationship-building. When understanding of communication shifts to encompass more than information exchange, we begin to recognize the role of communication in building relationships and influencing long term cultural shifts, which is often the goal for implementation scientists [3]. If the Process orientation is overlooked in favor of a Transaction orientation, we may miss opportunities for identifying evidence-based communication strategies to support implementation.

The majority of subsequent work engaging Manojlovich et al.’s assertions agree on the imperative to engage a Process orientation to communication, but they make no strides in designing approaches for exploring the characteristics of communication surrounding effective implementation strategies (e.g., [6–8]). As the conversation initiated by Manojlovich and colleagues about the role of communication in implementation science has progressed, recognition of communication has grown, but emphasis continues to focus on formal contexts (e.g., trainings and webinars) [1]. Further, quantitative measures that assess information accuracy like the one used in Zhao and colleagues’ work overlook the importance of informal communication (e.g., rapport-building before meetings, impromptu connections) and the nuanced influence of the relational dimension that contributes to effective implementation. Bustos et al.’s (2021) analysis acknowledges both the formal and informal strategies through which communication might occur, but the communication they refer to is discussed from a Transaction orientation (i.e., “how information… was communicated to program staff” (p. 10)) [9].

For this study, we draw on interviews with employees of the Department of Veterans Affairs (VA) who evaluated, implemented, and administered interventions focused on improving the health and well-being of rural Veterans or the clinical staff who serve them. These interviews were exploratory and wide-ranging; for the purposes of this manuscript, we treat the interviews as akin to direct observations of intervention stakeholders discussing their real-world experiences operationalizing implementation strategies. Instead of focusing on what we could learn from the communication described in the interviews, we directed our attention to what lessons could be missing because of the way participants discussed communication. In this manuscript, we provide examples of how Transaction and Process orientations to communication appear in the data when individuals described their experiences, as well as their relationships that supported IS strategies and facilitated intervention goals. We also suggest interview strategies to elicit detail about communication from a Process orientation to support ongoing learning of these informal communication processes. Though these interviews were not focused on communication, we use data from the interviews to argue that noticing communication helps us discover how to do implementation science better. Specifically, a Process orientation emphasizes the space between IS strategies and outcomes, and advances understanding of implementation challenges and solutions.

## Methods

### Study setting and context

The VA’s Office of Rural Health (ORH) supports the creation of Enterprise-Wide Initiatives (EWIs) to address issues facing rural Veterans from mental health and primary care access to training and education of VA staff who serve rural Veterans. As a part of the funding cycle, EWI teams must conduct annual evaluations. The Center for the Evaluation of Enterprise-Wide Initiatives (CEEWI) was created through a 2019 partnership between ORH and the VA’s Quality Enhancement Research Initiative to support EWI evaluation and disseminate best practices. The CEEWI team, consisting of implementation science experts and qualitative data analysts, reviews the annual reports and provides feedback to EWI teams on reporting standards.

#### Data collection

As part of the initial CEEWI project, EWI evaluators, implementors, and administrators were interviewed about effectiveness of IS strategies they used and why, in part, to assist the CEEWI team in understanding key aspects of EWI implementation and evaluation. The interview guide included questions about the participant’s role on the EWI, the core components of the EWI, implementation strategies and their impact on desired outcomes, outcome measures used for evaluation, and the evaluation process. CEEWI team members and EWI leadership identified the evaluators, implementors, and administrators to recruit for the study. While recruitment sought a purposive sample of roles from each EWI, ultimately the sample was a convenience sample based on availability and willingness to participate during the first nine months of the COVID-19 pandemic. Additional details about recruitment and data collection can be found in an earlier manuscript from this larger project [10]. We conducted 43 semi-structured interviews, which averaged 51 min (range 20–77 min), from April – December 2020 with evaluators, implementors, and administrators from 21 EWIs. While most interviews were conducted one-on-one, 8 were group interviews ranging from 2 to 4 participants [10]. This study uses these interviews as an example on how communication is described when discussing implementation strategies.

#### Data analysis

Audio-recordings were transcribed, reviewed for accuracy, and uploaded into MAXQDA, a qualitative data management software [11]. Two doctorally trained qualitative analysts (NJ & JVT) leveraged their previous IS knowledge and conducted primary-cycle inductive coding to identify IS constructs and trends in the data [12]. The analysts initially coded all transcripts together in real-time and resolved discrepancies immediately. During this first round of coding, several IS constructs were identified in participants’ discussion of their implementation strategies, including Staff Buy-in, Tailoring, Rapport, Fidelity, and Mentorship. Collaboration and Leadership Buy-in emerged as the two most discussed IS constructs among participants. For secondary-cycle deductive coding to interpret how communication was conceptualized in discussions of Collaboration and Leadership Buy-in, the lead author, a Health Communication scholar, used an iterative process to develop a codebook to identify the language representing a Process or Transaction orientation for each construct (i.e., Collaboration and Leadership Buy-in) [3, 12]. The analysis focused on the how communication was discussed, not about the form of communication that took place.

Collaboration, a term often characterizing various levels of formal and informal partnerships between individuals, departments or organizations, is defined as a mutually beneficial and well-defined relationship between two or more parties to achieve common goals [13]. An example of discussing Collaboration from a Transaction orientation to communication would be using the term *Collaboration* to describe monthly meetings where the parties update one another about the status of their tasks and goals. From a Process orientation, Collaboration would be discussed in relational terms, describing the trust and rapport the team members have among one another.

Leadership Buy-in represents the role of support from individuals in leadership positions for a program’s adoption and sustainability, particularly when competing clinical and administrative demands are at play [7]. An example of discussing Leadership Buy-in using a Transaction orientation to communication would be a description of strategies for adoption that only focused on leadership education. However, someone who engaged a Process orientation to communication might: (1) discuss tailored persuasive strategies for demonstrating value to specific decision-makers, or (2) acknowledge the necessity for long-term relationships with individuals in leadership roles for sustainment.

## Results

We conducted 43 interviews with 28 evaluators, 12 implementors, and 12 administrators. We coded a total of 90 segments as Collaboration (*n* = 46) and Leadership Buy-in (*n* = 44) across all the interviews. Most segments coded as Collaboration (*n* = 34, 74%) and Leadership Buy-in (*n* = 31, 70%) discussed communication from a Transaction orientation. The following results present examples of the discussion of Collaboration and Leadership Buy-in from the Transaction and Process orientations to communication.

### Transaction orientation to communication

When communication is treated as a transaction, it is discussed as a one-way flow of information traveling from one party to another during a discrete moment in time [3]. The materiality of communication is reduced to accurate, efficient information transfer, thus putting emphasis on the channel (e.g., Teams meeting, email) through which information is exchanged and the task dimension of the interaction.

### Collaboration as transaction

Participants sometimes discussed Collaboration in a way that missed its nuance and treated communication as merely a means for transferring information that produced Collaboration. For example, one participant implied that communication, regardless of quality, is inherently good, thus the more there is, the better. They identified “communication across the team level” as an important strategy having the most impact on desired outcomes. “The more communication there is, the more people are able (…) to divide up [responsibilities].” (1A) In this instance, communication is synonymous with information exchange. While we do not have enough information to assess the quality of communication that Participant 1A is referring to, the fact they only discussed the parties involved and quantity of communication is an example of the Transaction orientation to communication.

In another example, a participant explained what they felt did not work as well in their evaluation process. “We have excellent communication with some, but not all members of the [EWI] (…) I’m not sure they’re always on the same page with each other, and then depending on who we’re having a meeting with, we might hear one thing but then that’s not what someone else was going to do (…) that’s one of the pieces that I think is hard for us.” (2A) Again, we see the Transaction orientation, and the barometer for effective communication is accuracy. The participant went on to discuss ways to improve this lack of alignment among team members, suggesting that “even if it’s just being invited to join calls (…) [for us] to answer questions about the [evaluation] data” would improve teamwork. (2A) This passage highlights an important aspect of communication – being present for an interaction and having the opportunity to answer questions enables information exchange.

One participant described the communication that occurred during a monthly videoconference:

The learning collaborative is focused on bringing people [together] to share their experiences and how various facilitators identify ways to shape their program, but also the way that our national team gives feedback about the data (…) One call a month is right after a report (…) they do a data review on the call where they go over the numbers with the entire learning collaborative, everyone in the program, giving them feedback from a national perspective and always reminding people of the milestones of the metrics that they’ve agreed to under the ORH grant. (3A)

Here, we see another example of a participant discussing communication in terms of information exchange.

### Leadership buy-in as transaction

Participants also discussed Leadership Buy-in from a Transaction orientation. In the following passage, participant 4A described the benefits of the EWI leadership team visiting sites in-person:

They would do a site visit to all the hubs (…) and meet with the local leadership team and that’s where they confirmed if there were any issues that they might have. They would do like a 2–3 day site visit (…) so it helped create that structure where people knew exactly who to report to and how these programs were established and plenty of opportunities to address any concerns or any issues they might have.

There are substantial implications for local Leadership Buy-in through in-person visits, yet the only aspect of communication discussed here is information exchange and clarifying the information flow hierarchy (i.e., who to report to).

Participant 5A described their program’s efforts to obtain Leadership Buy-in:

Simple outreach and education, that was really the only things that we could do, and then as they continued, training kind of showed its usefulness. That had an impact on leadership buy-in.

Here, buy-in is attributed to education, which may account for some or even most of buy-in, but it does not recognize the relational dimension of communication.

For another EWI, leadership turnover at the facility presented a significant barrier to program sustainment, because Leadership Buy-in was perpetually reset, which exacerbated a “conflict between implementation and sustainment strategies” when the decision-maker for sustainment funding was not the same person to “sign off on it originally” (9A). Given the EWI provided seed-funding for specialty staff to implement the program, the expectation was that the facility would eventually incur the expense for sustainment, but the plan for funds was not made explicit at the time of application for the seed-funding. Participant 9A went on to explain how their program responded to the unforeseen challenge obtaining sustainment funding from sites:

Our clinical director worked really hard with the first cohort of sites prior to their funding ending to try to come up with strategies to pitch the program to leadership (…) Most sites had challenges with changing leadership priorities.

In response, the interviewer clarified their sources for funding, then changed topics: “Interviewer: Ok, alright. How about strategies that were intended to optimize the effectiveness outcomes for your EWI?” In this example, the interviewer seems to be approaching the participant’s description of Leadership Buy-in from a Transactional orientation. A Process-oriented approach that asked about the nature and details of pitching the EWI to leadership may have provided more information about implementation strategy.

### Process orientation to communication

From the process perspective, no single interaction serves as the cause or proof of effective Collaboration. Rather, the Process orientation recognizes the value of communication lies in the cumulative outcomes of consistent, often routine, interactions.

### Collaboration as process

Collaborations require shared responsibility, mutual authority, accountability, and sharing of resources and rewards for success [13]. Collaboration in implementation has focused on strategies to enhance partners’ ability to work together to achieve mutual benefits. We identified examples from participants discussing Collaboration with a Process orientation to communication. From these examples we see that Collaboration is seen as a product of long-term efforts to develop positive relationships and establish trust and autonomy to make one’s own decisions. Many participants recognized the uniqueness and value in reaching the point of Collaboration. For example, Participant 10A shared, “The partnerships, it’s like a very special kind of relationship–, where we have to trust them, we rely on each other, but we also need to be able to make independent decisions.” Participant 6A also recognized the importance of relationships, “I would say they’re collegial but they’re not fully collaborative (…) when they’re really more deeply integrated and their role is understood and recognized (…) they are more collaborative members.”

One participant on a different EWI echoed this sentiment that individuals’ intent and motivations for the work should extend beyond the assignment to be considered Collaboration, “It’s not just trying to check off a box (…) there truly is a passion behind it, on all of our parts, and that has been wonderful.” (7B) Recognizing others’ intent for their work allows one to acknowledge how interpersonal communication is influenced by more than information exchange.

In the following exemplars, we can see how interviewers were able to elicit detail about the interactions surrounding the implementation strategies they were discussing.

***Exemplar for Probing Collaboration***. In Table 1, we share an exemplar for engaging the Process orientation to communication, which led to greater explication of the role of communication in the implementation process.

**Table 1:** Engaging process orientation to communication while discussing collaboration

Exemplar of Interview Probing to Engage Process Orientation to Communication
In this example, Participant 9 A discussed a variety of IS strategies that impacted the EWI’s outcomes, which engaged the Process orientation to communication with the help of the interviewer’s follow-up questions:
Interviewer: Let’s return to the three strategies that you considered most impactful (…) the first strategy was facilitation. How would you define or describe it? Do you see that strategy as a discrete or single task or a complex and multifaceted strategy?
Participant: I think it’s multifaceted (…) Sometimes it’s taken the form of a nurse Skyping someone on our team to ask questions. Other times, phone calls. Other times, organized meetings with all of the nurses (…)
Interviewer: Is there more that you can say about what the key components or steps are for, for facilitation in the way that you used it?
Participant: I think the first step was just setting up a line of communication (…) and then I think being able to have open communication where you [have] non-judging communication where if there is a problem or if someone needed help, they didn’t feel uncomfortable asking for it. And I think a big part of it was being able to respond in a timely manner (…) So I think that consistency and responsiveness is really important.

Through this example, we see a more nuanced treatment of communication as a process after the interviewer probed twice to understand the participant’s use of “facilitation” as an implementation strategy. We gained description of the collaborative atmosphere within a team and how individuals’ psychological safety is manifested through authentic interactions.

### Leadership buy-in as process

It takes more than information-exchange to garner support (e.g., financial, staff) for facilitation and sustainment. One participant acknowledged the web of influence that contributes to Leadership Buy-in and effective implementation:

We reached out to all the rural sites their leadership… sort of advertising the program, so we would schedule a conference call with a director, chief of staff, emergency room chief, to sort of discuss the program (…) then we would follow up with an actual 1-day on-site visit (…) where we meet with again, leadership, but we also meet with the [staff from several departments] (…) It’s an all-day visit to further introduce our program, to the team on site, as well as learn more about their program, and how [our EWI] might incorporate itself, and what challenges (…) we might face in implementation. (2B)

Here, we see an acknowledgement of reciprocal relationship-building to learn about priorities and needs.

Several participants discussed how time costly it is to gain Leadership Buy-in to ease the burden of change on an organization and staff, particularly for a nationwide program. One participant reflected:

Ten years ago, it was a [regional] project, so the main kind of instruction came from a [regional] level down, you know. The site visit was just a medical director and the nurse manager telling you that, ‘Hey, this is what’s going to happen,’ and it happened. Now (…) it’s like a year-long process to get people familiarized (…) go live went from one day to four days long. (11B)

Despite its value, garnering Leadership Buy-in has its challenges. Sometimes identifying the right individuals who represent the relevant leadership roles is not clear cut.

Once we have identified that our program can go to that site, we ask the local (…) program manager to identify who (…) key local leaders are (…) It’s important to have the managers of those sites involved in this process from the beginning (…) We (…) set up an initial meeting (…) where we review the implementation process plan with everybody on that call, and answer questions about what we and [specialty care] services will provide as part of the training opportunity and clearly delineate what we need the site or the facility to commit to provide (…) we answer questions, alleviate concerns, things like that. (7B)

Participant 7B went on to describe the challenge of identifying the right leadership representative:

The only barrier that we’ve encountered is some challenges in getting the right leadership on the call to review this in real time and answer questions (…) whether it is due to leadership turnover at the site, even from the time that we set up the call to the time that we actually do the call, there have been some change-overs, and that has been a challenge.

Again, we see this participant engaging a strong Process orientation to communication as they emphasize the importance of relationship-building for Leadership Buy-in.

***Exemplar for Probing Leadership Buy-in***. In the following example, the interviewer engaged the Process orientation to communication with probes that led to greater explication of the role of communication in developing Leadership Buy-in (Table 2).

**Table 2:** Engaging process orientation to communication while discussing leadership buy-in

Exemplar of Interview Probing to Engage Process Orientation to Communication
Participant 5B explained sites had to provide letters of support from leadership at “different levels”:
So we made sure that we incorporated all of that by first introducing ourselves, the explanation of what we were doing (…) they included the (…) managers to get their buy-in. They did that by having the collaborative advisory board meetings (…) because all those players were present (…) The local educators would report to leadership their numbers and the impact (…) so that they were updated. They knew what was being presented when, how, and then they were given the results.
Interviewer: So leadership buy-in was kind of–, it seems like there were many different things that went into getting that?
Participant: You’re right, because it isn’t just, as we’ve learned, it isn’t just ‘Here, this is what we’re doing.’ If you get their collaboration again, the networking, all of that just kind of feeds into bringing in leadership and staff buy-in (…) And the advisory board was key, I think (…) and keeping an open network, you know, communication.

## Discussion

Results illustrate ways administrators, implementors, and evaluators characterized communication related to Collaboration and Leadership Buy-in. From the Transaction orientation, we saw that the term *communication* was used synonymously for information exchange. The problem of implementation lies beyond efficient and reliable information transfer, and instead centers on cooperative sensemaking and learning within and among teams situated in an organization that is influenced by its social, geographic, and political environments [2, 14, 15]. Communication necessary for effective implementation is based on improvisation and reciprocity and constitute relationships over time [2, 15]. Our data indicate these processes are occurring in implementation, but we may not always be paying close enough attention to their occurrence. If most discussions about communication engage a Transaction orientation, then practitioners and evaluators will never have the insight necessary to maximize the impact of their communication efforts.

Participants often discussed Leadership Buy-in more as an outcome of education, and less as a byproduct of improvisational relationship-building, which demonstrates the predominant Transaction orientation to communication privileging rehearsed, often unidirectional, and mostly controlled interactions. Formal information exchange is undoubtedly an important element of effective implementation; the Transaction orientation aligns well with the goals of dissemination and implementation as a field [15]. However, our data point to the importance of thinking about communication from a Process orientation for improving effectiveness of implementation strategies—and show how members of implementation and evaluation teams too often focus on the transaction elements of communication. Previous work that engages the Transaction orientation and points to the benefits of reliable information exchange has paved the way for more exploratory naturalistic methods for studying IS from a Process orientation to communication [3, 14–16]. As noted in our findings, the Transaction orientation overlooks the intricacies of processes that occur among individuals to build trust, cultivate buy-in, and influence team decision-making, all of which are markers of successful implementation.

### Suggestions for engaging process orientation to communication

Given the purpose of IS is to promote the adoption of research and evidence-based practices, it would behoove implementation scientists to tap into the richness of interdisciplinary theorizing and engage a Process orientation to communication [17]. As thinking about communication has evolved from a Transaction orientation, scholars recognized the symbolic process that humans use to create meaning through informal, improvised interactions over a period of time [2]. Recent analysis of implementation strategies for behavioral health interventions called for explicit attention to the supportive role communication may play in most, if not all, strategies [15]. The Process orientation to communication enriches theorizing and elevates scholars’ and practitioners’ understanding of how to leverage implementation strategies to be meaningfully responsive to the relationships among the interested parties [18]. However, we warn against over-characterizing communication into a ‘nebulous, global process’ [2, 19]. For gaining insight on communication processes, we suggest two strategies: 1) interviewers focused on understanding implementation strategies could probe their interviewees to learn more about how communication is enacted; and 2) IS practitioners could utilize process-oriented communication theories in developing interventions and evaluation tools (e.g., interview guides).

The supplementary material accompanying this article includes excerpts from our interview data as examples demonstrating hypothetical ways interviewers can elicit more nuanced understanding of communication processes (see Tables S1 and S2).

Our analysis identified examples of missed opportunities for interviewers to probe about communication from a Process orientation recognizing the relational dimension of communication. Interview probes like those recommended in Tables S1 and S2 could lead to valuable understanding of the processes of communication, allowing exploration of the relational dimension of communication and implementation, and insight to individuals’ attitudes and sensemaking about those experiences. This may contribute to a more nuanced understanding of the importance of communication in implementation strategies beyond a transactional information exchange. We also provided examples highlighting the constitutive role communication plays in relationship-building. Our goal is to help attune IS researchers to the value of the processes of communication as a critical component of many implementation strategies.

### Probing for communication processes in interviews

Challenges to implementing any new program may be significantly varied and widespread. No single barrier serves as an intervention’s fatal flaw, but rather, implementation is affected by numerous factors shaped through informal interactions [17, 20]. A recent study that aimed to identify which implementation strategies should be most closely considered for which determinants of practice reported one of its limitations was the heterogeneity of responses [21]. This variation in responses among administrators, implementors, and evaluators points to the value of a more nuanced understanding of the unique, context-dependent, and relationally based communication processes undergirding implementation strategies [21]. Further, in their ethnographic study on hand hygiene programs, Goedken and colleagues poignantly emphasized the importance of understanding *how* implementation strategies are used and defined in real-world settings for understanding determinants of practice [22]. By looking below the surface of implementation strategies and focusing on the interactions surrounding those strategies, we may begin to recognize the determinants of practices, the mechanisms for change, more precisely. Discussing communication from a Process orientation allows us to access what is happening below the surface that cannot be observed as an outsider. With greater insight on communication processes occurring throughout implementation, the field of IS would be poised to provide meaningful guidance for combining implementation strategies [22]. In a similar vein, IS researchers should consider the temporality of IS strategies and how this underscores the role of communication. The role of Leadership Buy-in at all stages of development and implementation on effectiveness cannot be overstated [23]. Albright suggests shifting away from the predominant focus of research on the active implementation period to explore activities occurring during design and preparation [15].

Most implementation strategies have a communication component representing the channel for education and promotion (e.g., workshops, webinars, brochures) [15]. Our proposed interview strategies interrogate communication in a way that recognizes the relational dimensions of interpersonal interactions, providing insight about what truly results in effective implementation. By understanding communication from a Process orientation, we may enrich our understanding of implementation strategies [24].

### Utilize process-oriented theories

Theories that engage a Transaction orientation to communication often ascribe to the traditional knowledge-intention-behavior paradigm that proposes a stable, linear positive relationship between knowledge and behavior change (e.g., Theory of Reasoned Action, a predictive theory suggesting a strong relationship among individuals’ attitudes about a behavior, their intention, and their behavior [25]) and tends to overlook the nuance of communication processes. However, humans are more complicated and inconsistent than these theories acknowledge. The Process orientation to communication allows for more realistic approaches that privilege the constitutive nature of communication to co-create meaning socially. In a recent scoping review of 158 studies in implementation research on maternity care, effective communication was noted as a key factor for promoting change across the body of work, but the majority of research was atheoretical and ambiguous in operationalization of communication [26].

Health communication scholars are trained to be sensitive to the cooperative nature of establishing shared meaning, multiple interpretations of behaviors, and the challenges of coordinating interactions when studying implementation strategies. Several theories, including two that pay special attention to how meaning is created socially, Coordinated Management of Meaning (CMM) [27] and Structuration Theory [28], could highlight perspectives that recognize communication as a complex process and translate well to practice. CMM is a constructivist theory that provides a practical heuristic for interpreting interpersonal communication events that comprise larger conversations. As such, CMM informs practitioners’ decision-making by illuminating patterns of interactions to find ways of talking that could result in desired outcomes [29]. Structuration Theory, coined by sociologist Anthony Giddens in the late 1970s, describes the dynamic relationship between individuals and their environment that constrains and enables social practices [28]. Through its critical lens, Structuration Theory highlights the (lack of) agency individuals perceive for themselves and others, and the rules and resources perpetuated through social interactions. Lastly, Diffusion of Innovations, a framework well-entrenched in IS research and practice, also engages a process paradigm [30, 31]. There is ample opportunity and an imperative to employ a Process orientation to better understand communication in implementation science.

### Limitations

This study has multiple limitations. We did not collection demographic data to describe our participants beyond the role they held on their EWI teams. The data represents a convenience sample of administrators, implementors, and evaluators working on EWIs funded at the time of data collection, which resulted in variability in representation across EWIs and staff roles. Further, because of the diversity of foci, designs, and timelines of EWIs, we cannot draw conclusions about effectiveness of strategies discussed in this paper. Lastly, the interviews were not conducted to assess communication explicitly. Despite these limitations, our analysis facilitates concrete suggestions for improving understanding of the role of communication in implementation.

### Future directions for research

Research analyzing the role of communication from a Process orientation would enrich the field of IS. Similar to Fishman et al.’s work comparing measurement and operationalization of attitude among IS studies and those grounded in psychology, our work emphasizes the importance of interdisciplinary collaboration [32]. The interviewees and interviewers in our study focused predominantly on a Transaction orientation to communication; more studies are needed that focus on this level of distinction, particularly how to adopt a Process orientation to communication for implementation strategy specification. There is great potential for a body of knowledge about communication processes that has been systematically developed to inform IS strategies supporting a range of aspects crucial to effectiveness including Leadership Buy-in and Collaboration. Future research may do well to conduct direct observation to characterize communication processes related to implementation strategies from a rich Process orientation. Dissemination Science, as one facet of Dissemination and Implementation Science, is firmly rooted in the mechanics of communication and would greatly benefit from engaging the Process orientation. A recent scoping review demonstrated that the field of Dissemination Science lacks insight to communication from the Process orientation; in their review of dissemination determinants, the Transaction orientation persists in focusing on imparting information from one party to the next [33].

## Conclusion

This study described instances of two broadly accepted orientations to communication engaged by implementation scientists. The findings demonstrate opportunities – and strategies – for engaging in the Process orientation of communication to gain greater insight into the role communication plays in implementation outcomes. We hope this work inspires dialogue, new interdisciplinary collaboration, and innovative methods to highlight the utility of engaging the Process orientation to communication to undergird the value of communication theory to implementation science for improving health services. When communication is understood as a process, practitioners will be better able to prepare for the unpredictability and uniqueness of the relational dimensions of communication.

## Supplementary Information

Supplementary Material 1. 

## Data Availability

The datasets presented in this article are not readily available in accordance with federal requirements and standards and guidelines for the protection of participants’ privacy and to maintain confidentiality. Requests to access the datasets should be directed to Dr. Heather Reisinger (heather.reisinger@va.gov).

## Abbreviations

CEEWI: Center for the Evaluation of Enterprise-Wide Initiatives
CMM: Coordinated Management of Meaning
EWI: Enterprise-Wide Initiative
IS: Implementation Science
ORH: Office of Rural Health
VA: Department of Veterans Affairs
