# Supplementary material for: Gaps in communication theory paradigms when conducting implementation science research: qualitative observations from interviews with administrators, implementors, and evaluators of rural health programs
Source: Implement Sci. 2024 Sep 16;19:66. doi: 10.1186/s13012-024-01395-3 (PMC11403836; doi:10.1186/s13012-024-01395-3)
Supplement: Supplementary file 1 — Supplementary Material 1. [file 13012_2024_1395_MOESM1_ESM.docx]

Additional File 1

For each IS construct, we use an example to suggest hypothetical interview probes for engaging the Process orientation to communication and gaining more nuanced understanding of the communication processes as they relate to implementation strategies.

**Opportunity for Probing Collaboration**

Interviews were not specifically designed to elicit detail on communication; however, opportunities arose to gain more information about communication from a Process orientation in order to better understand the effectiveness of implementation strategies. In Table S1, we present an example and outline potential probes to better elicit details of communication from a Process orientation.

| Table S1. Recommendation for Discussing Collaboration from a Process Orientation to Communication. |
| --- |
| **Example of Missed Opportunity to Probe Collaboration to Engage Process Orientation to Communication.** |
| One participant described effective Collaboration:  Participant: They have a required phone call once a week (…) there’s very good communication and kind of an opportunity to share difficult cases and ask for other people’s opinions. It’s very collegial (…) the [specialty care] providers feel that sense of collegiality, and (…) one of the providers said they felt actually more of a part of the team with the [remote program] providers than with the other [providers] that were in their hub facility.  Interviewer: So the different [specialty care] providers you’re talking about, where are they all located?  Participant: They’re all over the country (…)  Interviewer: It sounds like for each of these sites as far as maintenance goes, there’s not really anything that they have to do, there’s not like an FTE that they have to fund or anything? (2A)  This example demonstrates the participant’s attention to the way communication influences Collaboration, but the interviewer does not probe further. Here, we see an opportunity to learn what it meant to have “good communication” that enabled “collegial” relationships in a remote environment thus facilitating Collaboration. |
| **Recommended follow-up questions:**   - *Could you describe the collegial relationships you mention in more detail?* - *What do collegial relationships look like in your experience?* - *How did you feel during and after participating on the weekly phone calls?* - *What do you think led some providers to feel there was close Collaboration?* - *What do you think led to that sense of collegiality? Are there any specific examples of things that were said or done during those meetings that you could share?* |

**Opportunity for Probing Leadership Buy-in**

One implementor (6B) emphasized the role of consultative visits with prospective sites for obtaining Leadership Buy-in. The participant indicated their awareness of communication from a Process orientation, but the interviewer missed an opportunity to probe more about the details of the consultative visits. Table S2 outlines two parts of an interview and provides examples for eliciting more conversation about the processes of communication.

| Table S2. Recommendation for Discussing Collaboration from a Process Orientation to Communication. |
| --- |
| **Example of Missed Opportunity to Probe Leadership Buy-in to Engage Process Orientation to Communication** |
| Participant 6B recognized the importance of Leadership Buy-in to promote adoption and effectiveness. Because adoption and effectiveness were discussed as separate constructs at different points of the interview, we organize the following example in two parts:  **Part 1:**  Interviewer: Any other strategies that you could think of in regards to adoption?  Participant: Working closely with the leadership at the facility, because they’re really the part of the boots on the ground to implement this all. I think those monthly meetings really helped (…) to get everyone on the same page (…) We just needed to work closely with all those leaders of facilities.  Interviewer: Can you think of strategies that were specifically designed to help with implementation? |
| **Recommended follow-up questions (Part 1):**   - *What does it mean to “work closely” with leadership?* - *Tell me more about these monthly meetings. What did you plan for these meetings? What did a typical meeting look like?* - *Tell me more about the types of relationships “boots on the ground” have with one another.* - *In terms of adoption, what qualities in a leader encourage more staff buy-in?* |
| **Part 2:**  Interviewer: What three strategies do you feel had the most impact on the desired outcomes of the [EWI]?  Participant: I would say making sure that the leadership is engaged. We often go to them first about issues and if the leader is not as engaged (…) then it’s very difficult to implement a project (…) I think the consultative visits were really helpful (…) [and] gave us opportunities to talk and understand what was going on [at the site]. So talking with the chiefs of staff, or facility directors (…) we could help [specialty service] by being able to highlight some of these successes (…) that they may not have been able to otherwise get that attention or get that audience with facility leadership.  Interviewer: Okay, I guess I see a connection between these consultative visits and leadership engagement.  Participant: Yeah, definitely.  Interviewer: Of all these strategies, which three do you feel were used the most frequently?  Participant: The consultative visits would probably be the most impactful. As a one-time occurrence if a consultative visit occurred, then you should see the impact that it had on that facility and leadership.  Interviewer: What do you feel were the top three most feasible strategies used? |
| **Recommended follow-up questions (Part 2):**   - *Tell me more about these consultative visits. How could you tell that you were connecting with leadership?* - *What did you plan for the consultative visits? Is that typically what happened? Were there times when you had to improvise? If so, can you share an example?* - *It’s clear you see the consultative visits as valuable for Leadership Buy-in as it’s related to effectiveness. Can you explain a bit about your perspective on the role of Leadership Buy-in on the outcomes of the EWI?* - *Were there notable conversations you had with decision-makers that you felt were particularly impactful for Leadership Buy-in? If yes, please describe that experience.* |
